# Haemato-biochemical profile and acid–base status of Croatian spotted goats of different ages

**DOI:** 10.5194/aab-62-455-2019

**Published:** 2019-07-24

**Authors:** Zvonko Antunović, Ivica Marić, Željka Klir, Vatroslav Šerić, Boro Mioč, Josip Novoselec

**Affiliations:** 1 Faculty of Agrobiotechnical Sciences Osijek, J. J. Strossmayer University of Osijek, V. Preloga 1, 31000 Osijek, Croatia; 2 Croatian Agricultural Agency, Ilica 101, 10000 Zagreb, Croatia; 3 Department of Clinical Laboratory Diagnostics, University Hospital Osijek, J. Hutlera 4, 31000 Osijek, Croatia; 4 Department of Animal Science and Technology, Faculty of Agriculture, University of Zagreb, Svetošimunska cesta 25, Zagreb, Croatia

## Abstract

The aim of the present research was to determine the haemato-biochemical profile and blood acid–base status of Croatian spotted goats in a traditional Mediterranean production system. The 60 non-gravid female Croatian spotted goats of different ages were included in the research. They were divided into four groups of 15 goats according to age: group I – ≤1 year old; group II – 2–3 years; group III – 3–6 years; and group IV – 7–10 years. Haematological parameters were determined in whole blood, biochemical parameters in serum and acid–base status in plasma by automatic analyser. Total leukocyte number (WBC), haemoglobin (HGB) and mean corpuscular volume (MCV) in the blood were the highest, while mean haemoglobin concentration in erythrocytes (MCHCs) was the lowest in yearlings compared to other groups. Concentrations of urea, Mg, Cl, non-esterified fatty acids (NEFAs) and lactate were the highest in yearlings. Concentrations of Ca, Na, total cholesterol, high-density lipoprotein (HDL), very low-density lipoprotein (VLDL) and beta hydroxybutyrate (BHB) as well as the activity of alanine aminotransferase (ALT) were higher in older goats compared to yearlings, while the opposite was determined for the activities of creatine kinase (CK) and alkaline phosphatase (ALP). Values of pH, the strong ion difference (SID), anion gap (AG) and z values as well as the content of ${\mathrm{HCO}}_{3}$ and total pressure of carbon dioxide (${\mathrm{ctCO}}_{2}$) were higher in older goats compared to yearlings. The results obtained may help in monitoring the health and nutritional status and improve the management of Croatian spotted goats. Based on the results of the present study, the effect of age needs to be included in the model when preparing the reference values for the haemato-biochemical profile and acid–base status of goats.

## 1 Introduction

Determination of haemato-biochemical profile and acid–base status is very important for monitoring the metabolism and health status of small ruminants (Carlos et al., 2015; Antunović et al., 2017a, b). The values of haemato-biochemical parameters and acid–base indicators of goats are influenced by several factors such as breed, age, physiological status, sex, nutrition or season (Piccione et al., 2010; Arfuso et al., 2016; Antunović et al., 2017a; Redlberger et al., 2017). Biochemical parameters, primarily metabolites, enzymes, proteins and acid–base parameters of the blood, indicate possible metabolic disorders and disorders caused by inadequate nutrition (Antunović et al., 2002; Rios et al., 2006). In recent years, the determination of the acid–base status of blood has included more parameters such as the calculation of the anion gap (AG), strong ion difference (SID), z values, base excess, and the determination of organic acids (lactate, keto acids) and inorganic anions (sulfates, phosphates, etc.). The values of the acid–base status have not yet been determined for goats, and the precise negative charge of albumin and globulins and their dissociation constant is still unknown (Redlberger et al., 2017). Gärtner et al. (2019) suggested using more information about the acid–base status by calculating SID, weak anions and cations (ATOTs), the strong ion gap (SIG${}_{\mathrm{alb}}$), or unmeasured anions (XA) compared to traditional parameters. Besides, it is important to specify the exact values for every breed of goat of all ages, since climate zones and regions affect the haemato-biochemical profile and acid–base status of animals equally.

The Croatian spotted goat, known as a Balkan goat, is the most abundant goat breed in Croatia (estimated population is approximately 25 000 heads; CAA, 2018), developed and mostly reared in the karst, barren and inaccessible area of the Velebit, Dinara, Kamešnica and Biokovo mountains. Due to their skills and mobility, these goats are reared on the most inaccessible terrain, which is not adequate for rearing other livestock, especially cows (Mioč et al., 2008). The Croatian spotted goat is resistant and flexible with modest requirements for rearing and feeding. Production traits of an adult goat are as follows: average body weight is 44 kg, while the milk yield in 150–250 d of lactation is 100–250 L of milk (Mioč et al., 2008; Antunović et al., 2018). However, comprehensive research on the haemato-biochemical profile and the acid–base blood status of the Croatian spotted goat is missing. Therefore, the aim of the present research is to determine the haemato-biochemical profile and blood acid–base status of Croatian spotted goats in Mediterranean production systems.

## 2 Materials and methods

### 2.1 Animals and experimental design

Committee for Animal Welfare of Faculty of Agrobiotechnical Sciences Osijek established that the present research is carried out under the legal provisions according to Animal Protection Act (official gazette no. 133 since 2006, no. 37 since 2013 and no. 125 since 2013). Animal care and conditions of the research followed the recommendations of European Union directive 86/609/EEC (Council of the European Union, 1986).

The analysis of haemato-biochemical profile and blood acid–base status was carried out with 60 female Croatian spotted goats, which were of different ages and not gravid. All goats were reared in a traditional Mediterranean production systems. They were selected from a herd of 160 goats, before mating, while buck was not in the herd. The selected goats were healthy and in good physical condition. They were divided into four groups of 15 goats according to age as follows: group I – ≤1 year old (yearlings); group II – 2–3 years (young goats); group III – 3–6 years (adult goats); and group IV – 7–10 years (old goats). Goats were grazing extensive Mediterranean pastures from the early morning until 10:00 LT, when they returned to the stable, and were fed with hay (ad libitum) and approximately 0.2 kg d${}^{-1}$ of corn per head. Water and salt were offered to goats ad libitum.

The present study was carried out in October of the year 2017 when goats were not gravid. The family goat farm was located in Kuna Pelješka (latitude 42.967${}^{\circ }$ N, longitude 17.347${}^{\circ }$ E) 20 km from Orebić in Croatia. The monthly mean temperature for this area from October 2017 was 22 ${}^{\circ }$C, which is more than the standard average (from 1961 to 1990) of 17.9 ${}^{\circ }$C; the mean monthly rainfall was 50 mm, which is actually arid as compared to average values of 128.4 mm.

### 2.2 Analysis of feedstuffs

All feed samples (corn, hay and green forage from pastures) were dried and ground into a fine powder using a heavy metal free ultra-centrifugal mill (Retsch ZM 200) or knife mill (GM 200). The crude protein content of feed samples was determined by the Kjeldahl method (Pearson, 1976), while ether extract was determined according to the method described by Onwuka (2005). The crude fiber content was determined by the Weende method (Offor et al., 2014). Digestible and metabolisable energy of feed for goats were estimated according to DLG (1993). The concentrations of mineral elements in solutions of digested plant samples were determined by inductively coupled plasma (ICP, PerkinElmer Optima 2100 DV). All samples were analysed in duplicates. The chemical composition of feedstuffs for goats (mg kg${}^{-1}$) is presented in Table 1.

**Table 1:** Chemical composition of ingredients in goats' diets.

	Feedstuffs		
	Green	Hay	Corn
	forage		
Parameters, % DM			
Dry matter	22.70	91.13	87.02
Crude proteins	16.08	16.24	7.80
Crude fiber	25.20	35.56	2.44
Crude ash	9.46	7.49	1.09
Crude fat	3.54	2.32	3.02
Digestible energy, MJ kg${}^{-1}$ DM	2.31	8.65	14.77
Metabolisable energy, MJ kg${}^{-1}$ DM	1.87	7.09	12.11
Mineral content, mg kg${}^{-1}$ DM			
Ca	79.48	10 098.20	10 150.46
Mg	1594.66	3086.07	3477.02
K	5755.42	6387.98	14 922.26
P	4212.00	1162.38	2261.69
Na	68.03	1587.32	495.21
Fe	45.55	90.18	224.10

DM – dry matter.

### 2.3 Blood sample collection and parameters analysed

All blood samples were taken the same day, while goats were in the stable, after morning feeding. From each goat, three blood samples were collected from the jugular vein (10 mL) into sterile vacuum tubes (Venoject^®^; Sterile Terumo Europe, Leuven, Belgium). For haematology analysis, blood was collected into vacuum tubes containing ethylenediamine tetra-acetic acid (EDTA) as anticoagulant. Determination of haematological parameters (leukocyte number – WBC; erythrocytes – RBCs; haemoglobin – HGB; haematocrit – HCT; mean corpuscular volume – MCV; the average haemoglobin content in erythrocytes – MCHs; mean haemoglobin concentration in erythrocytes – MCHCs) in whole blood of goats was carried out on an automatic three-part differential haematology analyser Sysmex PocH-100iV (Sysmex Europe GmbH, Hamburg, Germany). Haematology analysis was carried out the same day after sampling. A differential leukocyte number test was carried out by microscope using the prepared blood smears coloured by Pappenheim.

Another blood sample was collected in sterile vacuum tubes, without anticoagulant, and centrifuged at 3000 revolutions per minute for 10 min and the obtained serum samples were frozen at -80 ${}^{\circ }$C and stored until analysis. In serum, concentrations of the following parameters were determined: calcium, inorganic phosphorus, potassium, sodium, magnesium, iron, chloride, urea, glucose, total proteins, albumin, creatinine, cholesterol, high-density lipoprotein (HDL) cholesterol, low-density lipoprotein (LDL) cholesterol, triglycerides (TGC), lactate, β-hydroxybutyrate (BHB) and non-esterified fatty acids (NEFAs). The activities of the following enzymes were also determined: alanine aminotransferase (ALT), aspartate aminotransferase (AST), alkaline phosphatase (ALP), creatine kinase (CK) and γ-glutamyl transferase (GGT). All aforementioned parameters were measured using Olympus System reagents (Olympus Diagnostic GmbH, Lismeehan, Ireland) by automatic analyser Olympus AU400. Globulin content was calculated as the difference between total protein and albumin. Very low-density lipoprotein cholesterol (VLDL) content was calculated as triglycerides divided by 5. The activity of glutathione peroxidase (GPx) in the serum was determined using a Ransel^®^ kit (Randox, UK) analyser, while the activity of total superoxide dismutase (SOD) in serum was determined with a Ransod^®^ kit (Randox, UK) on an automatic analyser (Olympus AU 400, Olympus, Japan).

Samples of plasma were obtained from sterile vacuum tubes containing Li-heparin, frozen at -80 ${}^{\circ }$C and stored until analysis. Plasma was analysed by automatic analyser Rapid Lab 348, which works on the basis of ion-selective electrodes. The following parameters were determined: pH, partial pressure of carbon dioxide ($p{\mathrm{CO}}_{2}$), partial pressure of oxygen ($pO_{2}$), total pressure of carbon dioxide ($t{\mathrm{CO}}_{2}$), actual base excess Cbase (B), standard base excess Cbase (Ecf) and electrolytes (${\mathrm{Na}}^{+}$, $K^{+}$, ${\mathrm{Cl}}^{-}$ and ${\mathrm{HCO}}_{3}^{-}$ – bicarbonate). SID was calculated following Eq. (1) according to Stewart (1983), the z value was calculated following Eq. (2) according to Whitehair et al. (1995) and the anion gap (AG) following Eq. (3) was calculated according to Kaneko et al. (2008):

1SID=Na++K+-Cl-,2zvalue=SID/Na,3AG=Na++K+-Cl-+HCO3-.

Weak anions and cations (ATOT${}_{\mathrm{tp}}$ and ATOT${}_{\mathrm{alb}}$) were calculated by multiplying the total protein (g/dL) or albumin by 2.9 (Constable, 1999; Waller and Lindinger, 2005). Strong ion gap (SIG) calculations were based on the serum concentrations of albumin (ATOT${}_{\mathrm{alb}}$, SIG${}_{\mathrm{alb}}$) and total proteins (ATOT${}_{\mathrm{tp}}$, SIG${}_{\mathrm{tp}}$) according to Gärtner et al. (2019) following Eqs. (4) and (5). In these calculations pKa = 7.06 was used according to Constable (2002). Following Eq. (6) by Gärtner et al. (2019) unmeasured anions (XA) were calculated:

4SIGalb=ATOTalb/1+10(pKa-pH)-AG,5SIGtp=ATOTtp/1+10(pKa-pH)-AG,

6 $$\begin{array}{rl} \mathrm{XA} & =c{\mathrm{Na}}^{+}+cK^{+}+c{\mathrm{Ca}}^{2+}+c{\mathrm{Mg}}^{2+}-c{\mathrm{Cl}}^{-}-c{\mathrm{HCO}}_{3}\\ & -[0.141\times \;\mathrm{albumin}\;\times (\mathrm{pH}-5.42)]\\ & -[0.04\times \;\mathrm{globulin}\times (\mathrm{pH}-5.58)]\\ & -[\mathrm{phosphate}\;\times 0.309\times (pH-0.469)]. \end{array}$$

### 2.4 Statistical analysis

The results of goat's blood regarding the concentration of biochemical parameters, electrolytes, enzymes activities and indicators of the acid–base balance were obtained by the MEANS procedure, and presented as mean value and standard error of mean. Analysis of variance was performed with general linear model (GLM) procedure with age as fixed effect, while differences between groups were determined by a Tukey test at the level of P<0.05. All data were analysed with the statistical software SAS 9.4^®^ (SAS Institute Inc., 2002–2012).

## 3 Results

Analysing Table 2 it is evident that the WBC number and MCV in the blood of goats from group I was higher compared to groups of older goats. Haemoglobin in group I was higher compared to groups II–IV, while in group IV, it was higher compared to group II. By contrast, the MCHC content was higher in older goats compared to group I. No differences were observed among groups II–IV. The distribution of leukocytes did not differ between goats of different ages.

**Table 2:** Haematological parameters and leukocyte distribution from goats of different ages.

	Group I	Group II	Group III	Group IV	SEM	P value
	(≤1 year)	(2–3 years)	(4–6 years)	(7–10 years)		
WBC, $\times 10^{9}$ L	16.28${}^{a}$	8.98${}^{b}$	8.89${}^{b}$	8.61${}^{b}$	0.58	<0.001
RBC, $\times 10^{12}$ L	13.32	11.90	12.10	13.31	0.21	0.050
HGB, g L${}^{-1}$	104.93${}^{a}$	87.07${}^{\mathrm{bd}}$	92.57${}^{b}$	95.43${}^{\mathrm{bc}}$	1.27	<0.001
HCT, L L${}^{-1}$	0.58	0.46	0.41	0.44	0.03	0.350
MCH, pg	7.89	7.33	8.59	7.19	0.33	0.436
MCV, fL	62.71${}^{a}$	39.15${}^{b}$	33.69${}^{b}$	33.74${}^{b}$	3.08	<0.001
MCHC, g L${}^{-1}$	156.13${}^{b}$	226.20${}^{a}$	238.85${}^{a}$	235.43${}^{a}$	9.99	0.006
Leukocyte distribution, %						
Lymphocytes	70.87	67.20	68.93	62.86	1.58	0.333
Neutrophils	28.47	31.07	29.21	35.50	1.52	0.376
Band cells	0.00	0.07	0.00	0.07	0.02	0.575
Eosinophils	0.60	1.60	1.85	1.50	0.17	0.055
Basophils	0.07	0.07	0.14	0.07	0.04	0.870

SEM – standard error of mean; a–d – values in rows with different letters differ significantly (P<0.05); WBC – leukocyte number; RBC – erythrocytes; HGB – haemoglobin; HCT – haematocrit; MCV – mean corpuscular volume; MCH – average haemoglobin content in erythrocytes; MCHC – mean haemoglobin concentration in erythrocytes.

**Table 3:** Blood biochemical parameters from goats of different ages.

	Group I	Group II	Group III	Group IV	SEM	P value
	(≤1 year)	(2–3 years)	(4–6 years)	(7–10 years)		
Urea, mmol L${}^{-1}$	9.23${}^{a}$	4.46${}^{b}$	4.70${}^{b}$	4.87${}^{b}$	0.36	<0.001
Total proteins, g L${}^{-1}$	80.34	83.98	83.06	86.46	1.05	0.215
Albumin, g L${}^{-1}$	32.5	33.74	33.06	32.79	0.32	0.582
Globulin, g L${}^{-1}$	47.83	50.24	50.00	53.68	0.98	0.197
A / G	0.69	0.68	0.67	0.63	0.01	0.266
Glucose, mmol L${}^{-1}$	2.18	2.28	2.52	2.73	0.10	0.173
Ca, mmol L${}^{-1}$	2.28${}^{b}$	2.73${}^{a}$	2.55${}^{a}$	2.55${}^{a}$	0.04	<0.001
P, mmol L${}^{-1}$	2.70	2.43	2.41	2.25	0.07	0.168
Na, mmol L${}^{-1}$	140.60${}^{b}$	144.73${}^{a}$	143.57${}^{\mathrm{ab}}$	145.00${}^{a}$	0.46	<0.001
K, mmol L${}^{-1}$	3.18	3.86	3.89	3.83	0.12	0.111
Mg, mmol L${}^{-1}$	1.10${}^{a}$	0.86${}^{b}$	0.84${}^{b}$	0.87${}^{b}$	0.02	<0.001
Cl, mmol L${}^{-1}$	109.47${}^{a}$	108.53${}^{\mathrm{ab}}$	105.57${}^{b}$	107.31${}^{\mathrm{ab}}$	0.46	0.018
Fe, µmol L${}^{-1}$	20.21	25.78	24.66	23.22	0.78	0.065
CHOL, mmol L${}^{-1}$	1.80${}^{a}$	2.00${}^{\mathrm{ab}}$	2.20${}^{\mathrm{ab}}$	2.25${}^{b}$	0.07	0.050
HDL, mmol L${}^{-1}$	1.03${}^{b}$	1.42${}^{a}$	1.39${}^{a}$	1.59${}^{a}$	0.04	<0.001
LDL, mmol L${}^{-1}$	0.65	0.54	0.48	0.53	0.03	0.238
VLDL, mmol L${}^{-1}$	0.04${}^{b}$	0.07${}^{a}$	0.06${}^{\mathrm{ab}}$	0.07${}^{a}$	0.01	0.002
TGC, mmol L${}^{-1}$	0.20${}^{b}$	0.34${}^{a}$	0.29${}^{\mathrm{ab}}$	0.35${}^{a}$	0.02	0.002
Lactate, mmol L${}^{-1}$	7.84${}^{a}$	5.13${}^{b}$	4.06${}^{b}$	4.87${}^{b}$	0.34	<0.001
NEFAs, mmol L${}^{-1}$	0.40${}^{a}$	0.14${}^{b}$	0.17${}^{b}$	0.19${}^{\mathrm{ab}}$	0.03	0.008
BHB, mmol L${}^{-1}$	0.13${}^{b}$	0.31${}^{\mathrm{ab}}$	0.34${}^{\mathrm{ab}}$	0.38${}^{a}$	0.04	0.047

SEM – standard error of mean; a, b – values in rows with different letters differ significantly (P<0.05); A / G – albumin / globulin; CHOL – cholesterol; HDL – high-density lipoprotein; LDL – low-density lipoprotein; VLDL – very low-density lipoprotein cholesterol; TGC – triglycerides; NEFAs – non-esterified fatty acids; BHB – β-hydroxybutyrate.

**Table 4:** Blood enzymes activities from goats of different ages.

	Group I	Group II	Group III	Group IV	SEM	P value
	(≤1 year)	(2–3 years)	(4–6 years)	(7–10 years)		
AST, U L${}^{-1}$	93.91	114.27	119.70	118.89	3.61	0.050
ALT, U L${}^{-1}$	14.23${}^{b}$	29.49${}^{a}$	31.22${}^{a}$	29.49${}^{a}$	1.28	<0.001
GGT, U L${}^{-1}$	44.18	44.15	44.07	44.67	1.28	0.999
CK, U L${}^{-1}$	290.67${}^{a}$	172.93${}^{b}$	181.14${}^{b}$	160.31${}^{b}$	10.97	<0.001
ALP, U L${}^{-1}$	298.37${}^{a}$	250.39${}^{a}$	246.20${}^{a}$	139.50${}^{b}$	31.26	0.047
SOD, U mL${}^{-1}$	0.63	0.45	0.45	0.54	0.03	0.092
GPx, U L${}^{-1}$	1240.55	1177.47	1117.57	1017.37	45.98	0.071

SEM – standard error of mean; a, b – values in rows with different letters differ significantly (P<0.05); AST – aspartate aminotransferase; ALT – alanine aminotransferase; GGT – γ-glutamyl transferase; CK – creatine kinase; ALP – alkaline phosphatase; SOD – superoxide dismutase; GPx – glutathione peroxidase.

**Table 5:** Blood acid–base balance from goats of different ages.

	Group I	Group II	Group III	Group IV	SEM	P value
	(≤1 year)	(2–3 years)	(4–6 years)	(7–10 years)		
pH	7.23${}^{b}$	7.38${}^{a}$	7.41${}^{a}$	7.42${}^{a}$	0.01	<0.001
$p{\mathrm{CO}}_{2}$, kPa	12.90${}^{a}$	10.71${}^{b}$	10.23${}^{b}$	9.68${}^{b}$	0.24	<0.001
$pO_{2}$, kPa	10.74	10.40	9.74	9.65	0.35	0.643
${\mathrm{HCO}}_{3}$, mmol L${}^{-1}$	24.41${}^{b}$	28.60${}^{a}$	29.24${}^{a}$	28.34${}^{a}$	0.40	<0.001
${\mathrm{ctCO}}_{2}$, mmol L${}^{-1}$	27.35${}^{b}$	31.05${}^{a}$	31.61${}^{a}$	30.51${}^{a}$	0.40	<0.001
Cbase (B), mmol L${}^{-1}$	$-8.53^{b}$	$-1.09^{a}$	$-0.39^{a}$	$-1.04^{a}$	0.58	<0.001
Cbase (Ecf), mmol L${}^{-1}$	$-5.33^{b}$	0.92${}^{a}$	2.10${}^{a}$	1.23${}^{a}$	0.53	<0.001
$sO_{2}$ %	82.78	78.27	86.54	87.13	0.94	0.282
SID, mmol L${}^{-1}$	34.31${}^{b}$	40.06${}^{a}$	41.89${}^{a}$	41.53${}^{a}$	0.61	<0.001
AG, mmol L${}^{-1}$	9.90${}^{b}$	11.46${}^{\mathrm{ab}}$	12.65${}^{\mathrm{ab}}$	13.23${}^{a}$	0.46	0.049
z values	0.24${}^{b}$	0.28${}^{a}$	0.29${}^{a}$	0.29${}^{a}$	0.004	<0.001
ATOT${}_{\mathrm{tp}}$, mmol L${}^{-1}$	27.56	28.81	28.49	29.66	0.36	0.215
ATOT${}_{\mathrm{alb}}$, mmol L${}^{-1}$	20.22	20.99	20.57	20.39	0.20	0.582
SIG${}_{\mathrm{alb}}$, mmol L${}^{-1}$	-0.11	0.45	-0.64	-1.22	0.49	0.662
SIG${}_{\mathrm{tp}}$, mmol L${}^{-1}$	3.39	4.89	3.98	4.22	0.52	0.798
XA, mmol L${}^{-1}$	3.94	0.86	2.35	2.32	0.43	0.092

SEM – standard error of mean; a, b – values in rows with different letters differ significantly (P<0.05); $p{\mathrm{CO}}_{2}$ – partial pressure of carbon dioxide; $pO_{2}$ – partial pressure of oxygen; ${\mathrm{HCO}}_{3}$ – bicarbonate; ${\mathrm{ctCO}}_{2}$ – total pressure of carbon dioxide; Cbase (B) –actual base excess; Cbase (Ecf) – standard base excess; SID – strong ion difference; AG – anion gap; ATOT${}_{\mathrm{tp}}$ – weak anions and cations multiplying the total protein by 2.9; ATOT${}_{\mathrm{alb}}$ – weak anions and cations multiplying the albumin by 2.9; SIG${}_{\mathrm{alb}}$ – strong ion gap based on the serum concentrations of albumin; SIG${}_{\mathrm{tp}}$ – strong ion gap based on the serum concentrations of total protein; XA – unmeasured anions.

Significantly lower concentrations of urea, Mg, Cl and lactate as well as lower activities of ALP and CK in goats' blood were observed in older goats compared to group I (Table 3). Significantly higher concentrations of Ca, Na, total cholesterol, HDL cholesterol, VLDL cholesterol and BHB as well as higher activity of ALT in the blood of older goats were observed compared to group I (Tables 3 and 4). The concentration of Na, VLDL and TGC did not differ when group III was compared with other groups. The concentration of Cl did not differ when group I was compared with group II and group IV. The cholesterol concentration was lower in group I compared to group IV, while groups II and III did not differ. The concentration of NEFA was higher in group I than in groups II and III, while the concentration of BHB was lower compared to group IV. The activity of SOD and GPx did not differ when comparing young with older goats, although a tendency of decrease with age was observed.

Analysing Table 5 in the blood of goats, lower pH, SID, AG and z values as well as the content of ${\mathrm{HCO}}_{3}$ and ${\mathrm{ctCO}}_{2}$ was determined in group I compared to older goats. By contrast, significantly higher $p{\mathrm{CO}}_{2}$, Cbase (B)e and Cbase (Ecf)c in the blood of older goats compared to group I was observed. The values of AG did not differ when comparing group I or group IV with groups II and III. The values of $pO_{2}$, $O_{2}$, ATOT${}_{\mathrm{tp}}$, ATOT${}_{\mathrm{alb}}$, SIG${}_{\mathrm{tp}}$, SIG${}_{\mathrm{alb}}$ and XA did not differ between groups.

## 4 Discussion

The majority of the results in the blood of haemato-biochemical parameters and acid–base indicators were within reference values (Kaneko et al., 2008; Antunović et al., 2013, 2017a). The haematological blood parameters, such as RBC, WBC and HGB, can reflect physiological occurrences in an animal's body. In the present study the WBC number, HGB concentration and MCV in the blood of goats was lower and MCHC was higher in older goats compared to group I. Arfuso et al. (2016) in a study with Messinese goats determined more RBC and a higher content of HGB in younger goats compared with older goats, as well as similar difference in MCV and the opposite trend in MCHC in the blood of Argentata dell'Etna goats. Similar results with RBC and HGB concentration in the same goat breed determined Piccione et al. (2010). The differences in RBC could be related to the oxygen-carrying capacity of the blood, which is higher in young goats compared with old ones (Daramola et al., 2005), while changes in WBC were connected with suitable protection systems, due to immune variations in animals (Piccione et al., 2014; Arfuso et al., 2016). The MCV and MCHC in goat fluctuated mostly depending upon RBC, HGB and HCT values. This is in accordance with conclusions by Egbe-Nwiyi et al. (2000). Generally, most of the haematological parameters determined in our study were within the normal range for goats reported by other authors (Zumbo et al., 2011; Habibu et al., 2017).

In the current study significantly lower urea, Mg, Cl and NEFA concentrations in goats' blood were observed as well as lower activities of ALP and CK, while significantly higher Ca, Na, total cholesterol, HDL cholesterol, VLDL cholesterol and BHB concentrations as well as higher activity of ALT was observed in older goats compared to group I. The results from the present study (4.46–9.23 mmol L${}^{-1}$) were within reference values for urea (2.9–10.9 mmol L${}^{-1}$,; McDougall et al., 1991) which may be related to the good content of proteins in goats' diet (Table 1). In fact, Kohn et al. (2005) showed that the concentration of urea can be considered as a good indicator of the amount of nitrogen consumed through feed. Piccione et al. (2010) obtained a decreased urea concentration in the blood of Girgentana goats from 1–2 to 3–4 years and an increased concentration from 5 to 6 years. An increase in urea concentrations in young non-pregnant goats was observed compared with adult Danish landrace dairy goats (Mbassa and Poulsen, 1991a).

In the present study, plasma Ca levels in goats younger than 1 year were at the limit of the reference values (Kaneko et al., 2008; 2.3–2.9 mmol L${}^{-1}$). Table 1 presents adequate concentrations of Ca in feedstuffs, which was offered to goats of all ages. A higher sodium concentration in the blood of older compared to younger sheep was determined Antunović et al. (2004). Gwaze et al. (2012) determined significantly lower concentrations of P as well as lower activity of ALP in older compared to young Nguni goats from South Africa. A lowered concentration of P in the blood could be the cause of a decreased capacity to assimilate phosphorus from the diet with increasing age of animals (Blood and Radostis, 1993). Kaneko et al. (2008) reported that serum phosphate may be higher in younger animals because the growth hormone increases renal phosphate resorption. In the present study the concentration of Mg was lower in older groups compared to group I. A similar trend for Mg concentration in the blood of Angora goats (1.66–1.44 mmol L${}^{-1}$) was determined by Van Niekerk and Cloete (1990) and in the research by Pandey et al. (2006) in Marwari goats (1.06–1.027 mmol L${}^{-1}$). As reported by Bhattacharyya et al. (1994) that was associated with an increased mobilisation of minerals from the bone because of an increased demand for growth at this stage or due to an increased rate of resorption from the gastrointestinal tract.

Higher concentrations of total cholesterol, HDL cholesterol and VLDL cholesterol in the blood of older goats were observed compared to group I. A similar trend for blood cholesterol concentrations was determined in Marwari goats from India (Pandey and Sareen, 2007) and in Hair goats from Turkey (Karaşahin et al., 2018). Very few studies are available regarding NEFA concentrations within goats' blood. Higher NEFA in yearlings compared to older goats indicates a certain energy deficit, since, according to Dunshea and Bell (1989), a concentration of 0.21 mmol/L is suggested for lactating goat at zero energy balance. Mondal and Prakash (2004) explained that the concentration of the growth hormone in the plasma of buffaloes decreases along with lipolysis resulting in less NEFA in the plasma as they become older. An increase in BHB in goat blood determined with increased age was within the physiological range, since Doré et al. (2015) reported a hypoketonemia definition based on BHB concentrations from ≥0.4 to ≥0.9 mmol L${}^{-1}$. According to Herdt (2000) BHB is more likely a reflection of nutritional status and less constrained physiologically.

In the present study, higher activities of ALT and AST in the blood of older goats may be used as indicators of physical stress (Mbassa and Poulsen, 1991b). The activities of ALT and AST in the blood of Girgentana goats increased with their age, which was determined by Piccione et al. (2010). Perez et al. (2003) determined that levels of ALP are the highest in young Spanish ibex compared to adult ones; Antunović et al. (2004) also determined levels in Merinolandschaf sheep which may have been correlated with high osteoblastic activity in young animals (Durak et al., 2015). The highest CK activity in the blood of young goats could be an indicator of specific markers of damaged muscle. Similar results were obtained in Merino landschaf ewes by Antunović et al. (2004). The increasing CK activity in calves could be attributed to their growth and gaining muscle mass (Klinkon and Ježek, 2012). Besides, Mpkama et al. (2014) determined higher CK activity in 18-month-old cattle compared to 16- or 24-month-old cattle. A tendency of SOD and GPx activity decreased with age was found in serum. Dubreuil et al. (2005) observed a decrease in GPx in the serum of ewes with age when comparing yearlings and ≥4-year-old ewes.

In a general sense, metabolic activity modifies the acid–base balance, although it is difficult to estimate the degree of this contribution to the metabolic component of the acid–base balance (Castillo et al., 2000). According to Kaneko et al. (2008), the recommended value for the anion gap in goats is 10–20 mmol L${}^{-1}$, but for SID values, it is 40.00 mmol L${}^{-1}$ (Castillo et al., 2000). In the present study an increase in pH values with increased age is due to a decrease in $p{\mathrm{CO}}_{2}$ (12.9–9.68 kPa) and ${\mathrm{ctCO}}_{2}$ concentrations. A similar situation was observed by Redlberger et al. (2017) in study with goats aged 6–56 weeks. Significantly lower pH in the blood of the youngest compared to older goats in this study is related to significantly lower ${\mathrm{HCO}}_{3}$, Cbase (B) and Cbase (Ecf). Afterwards, pH values as well as ${\mathrm{HCO}}_{3}$, ${\mathrm{ctCO}}_{2}$, AG, SID and z values were significantly higher in older goats. The anion gap is an indicator used to investigate the presence of unmeasured anions and may differ with $p{\mathrm{CO}}_{2}$ and ${\mathrm{HCO}}_{3}$ (Fencl and Leith, 1993; Castillo et al., 1998). However, the value of the anion gap is limited for an explanation of the acid–base balance. The ketone bodies, classified as unmeasured anions, were detected by increased XA and/or decreased SIG${}_{\left(\mathrm{Alb}\right)}$ and SIG${}_{\left(\mathrm{Prt}\right)}$ in the study by Gärtner et al. (2019), although these parameters did not differ between groups of goats aged differently in the current study. Therefore, in the correct interpretation of the acid–base status of goats, during different ages, SID, base excess and z values need to be incorporated.

## 5 Conclusion

The haemato-biochemical profile and acid–base status of Croatian spotted goats from the present study are the first published reference values and can be useful for understanding the metabolic profile of this breed. Meanwhile, in preparing the reference values for the haemato-biochemical profile of Croatian spotted goats, the inclusion of the age effect in the model is mandatory. Goats from 2 to 7 years or older mostly do not have a changed metabolic profile. When comparing the youngest goats with older ones, it is important to include certain biochemical parameters, enzyme activities and parameters of the acid–base status. The results obtained may help in monitoring the health and nutritional status of goats, while the implementation of these parameters provides more in-depth information regarding the metabolic profile of differently aged goats.

## Data Availability

The data sets are available upon request from the corresponding author.
